# Visuospatial ability and attention as risk factors for suicidal ideation in middle-aged and elderly schizophrenia patients: a cross-sectional study

**DOI:** 10.1186/s12888-023-05272-z

**Published:** 2023-10-18

**Authors:** Qiongzhang Wang, Wei Tang, Junjie Zhang, Yiwei Wang, Qing Wang, Yimin Ma, Jian Kai Mao, Chengyu Ye, Xin Yu

**Affiliations:** 1 School of Mental Health, Wenzhou Medical University, Wenzhou, China; 2 The Affiliated Kangning Hospital of Wenzhou Medical University Zhejiang Provincial Clinical Research Center for Mental Disorder, Wenzhou, China; 3 Tongde Hospital of Zhejiang Province, Hangzhou, China; 4 Wenzhou Central Hospital, The Dingli Clinical Institute of Wenzhou Medical University, Wenzhou, China; 5 Peking University Institute of Mental Health (Sixth Hospital, Beijing, China; 6 National Clinical Research Center for Mental Disorders and Key Laboratory of Mental Health, Ministry of Health, Peking University, Beijing, China; 7 Beijing Municipal Key Laboratory for Translational Research on Diagnosis and Treatment of Dementia, Beijing, China

**Keywords:** Age, Cognitive impairment, Insomnia symptoms, Positive symptoms, Schizophrenia, Suicidal ideation

## Abstract

**Background:**

Schizophrenia patients have a high risk of suicide, and their cognition function is impaired with increasing age. The association between neurocognitive and suicidality in schizophrenia patients are heterogeneous. We aimed to explore the relationship between neurocognitive function and suicidal ideation in schizophrenia patients across age groups.

**Methods:**

A total of 587 patients with schizophrenia were enrolled in this study. The schizophrenia patients were divided into young group (aged 18–44) and middle-aged and elderly group (aged 45–70). The schizophrenia patients were divided into suicidal ideation group and non-suicidal ideation group according to the evaluation results of the Beck Scale for Suicide Ideation. Insomnia symptoms were measured by the Insomnia Severity Index (ISI). Psychotic symptoms were measured by the Positive and Negative Syndrome Scale (PANSS), and cognitive function was measured by the Repeatable Battery for the Assessment of Neuropsychological Status (RBANS).

**Results:**

There was a negative correlation between the age and attention scores of RBANS (P = 0.018). The young schizophrenia patients had higher risk of suicidality than middle-aged and elderly schizophrenia patients (P = 0.001). In the logistic regression analysis, the scores of ISI and positive symptoms scores of PANSS were associated with suicidal ideation among young schizophrenia patients (All P < 0.05). Age, BMI, the scores of ISI, general symptoms scores of PANSS, visuospatial scores of RBANS and attention scores of RBANS were associated with suicidal ideation in middle-aged and elderly schizophrenia patients (All P < 0.05).

**Conclusions:**

High visuospatial scores of RBANS and attention scores of RBANS were risk factors for suicidal ideation in middle-aged and elderly schizophrenia patients.

## Introduction

Schizophrenia (SZ) is a common mental disorder which has a prevalence of approximately 0.28% [1]. SZ patients usually have a higher rate of suicide [2] than the general population. The lifetime prevalence of suicide in individuals with SZ ranges from 10 to 50% [3]. SZ is estimated to reduce life expectancy by 10 to 20 years, and suicide is the biggest single cause of premature death that contributes to this shortened life span [4, 5]. Suicidal ideation is a significant predictor of suicide attempt [6]. Suicidal ideation among patients with SZ results in a 20-fold greater suicide rate than that observed in the general population [7]. Suicidal ideation seriously harms the patient’s quality of life [8]. Given that China has the largest number of SZ patients in the world [9], how to properly treat and care for these patients with suicidal ideation is meaningful.

Suicidality is a complex social phenomenon, which is closely related to sociological, psychological, and biological factors. The risk factors included demographic variables(such as younger age, male [10], and higher premorbid IQ [11]), clinical characteristics (such as chronic disease courses, younger age at onset, increased positive symptoms, decreased negative symptoms [12], insomnia symptoms [13], a greater level of insight [14] ,and fewer cognitive deficits [15]), sociological factors(such as poor social function, poor social support [16, 17]), and psychological factors (such as emotional problems, hopelessness (16), and psychological pain [18]).

Neurocognition is a basic function of the central nervous system, including a multitude of cognitive domains such as executive function, working memory, process speed, attention, and episodic memory [19]. Neurocognition impairment is an essential feature of SZ [20]. Previous studies have reported that preserved neurocognitive function are related to suicidality in SZ patients [15, 21, 22]. However, other studies reported that suicidality is not correlated with neurocognitive function in SZ patients [23–25]. The association between distinct neurocognitive domains and suicidality in SZ patients are largely inconclusive [15, 21, 22]. A study carried on outpatients with SZ has showed that attention, memory and verbal fluency are associated with suicidal ideation [26]. Delaney et al. has reported that attention and memory are associated with suicidal attempt [15]. Zhang et al. has found that only attention is associated with suicidal attempt in first-episode and drug-naive patients with SZ [21]. There are various reasons for these heterogeneous results among the previous studies, such as patient selection bias, different age, different definitions of suicidal categories and different dosage of antipsychotics. Most studies explored the relationship between neurocognition and suicidal attempt [15, 21], while few studies have been done to explore the correlation between neurocognition and suicidal ideation in SZ inpatients.

The neurocognitive impairment in SZ patients is common with the increasing age [20]. Previous study has reported that neurocognitive function is correlated negatively with age in SZ patients [27]. No significant difference is found between the SZ and normal comparison groups in slopes that depicted age-related variation [28]. Specifically, compared to controls, SZ patients has showed greater deteriorated performance with increasing age on attention and greater slowness for processing social information rather than working memory and verbal memory [29]. No study has been done to explore the relationship between distinct cognitive domains and suicidal ideation in SZ patients across age groups.

Given that there is no further study focused on the relationship between distinct cognitive domains and suicidal ideation in SZ patients across age groups., we aimed to explore the relationship between distinct cognitive domains and suicidal ideation in SZ patients across age groups. It might help us clarify the relationship between distinct cognitive domains and suicidal ideation in SZ patients across age groups.

## Materials and methods

### Participants

This is a cross-sectional descriptive and analytical study. Patients who were independently diagnosed with SZ by two psychiatrists at the Affiliated Kangning Hospital of Wenzhou Medical University were included in our study from December 2018 to December 2019. All patients were assessed by Structured Clinical Interview for DSM-IV (SCID). All patients were recruited from inpatients. The clinical research described in the manuscript was carried out in accordance with the Declaration of Helsinki. This study was approved by the Medical Ethics Committee of the Affiliated Kangning Hospital of Wenzhou Medical University. The informed consents were signed by the patients and their relatives. All patients met the inclusion criteria: (1) A diagnosis of SZ according to the Diagnostic and Statistical Manual of Mental Disorders, Fourth Edition (DSM-IV), (2) Age 18–70 years, (3) At stable phase of SZ, (4) Han Chinese ethnicity. The exclusion criteria: (1) Patients with a concomitant physical disease severe enough that they could not complete the measurements (e.g. cardiovascular, liver, kidney, gastrointestinal diseases, epilepsy or head injury, etc.), (2) The female patients who are pregnant, planning to become pregnant, or breastfeeding during the study period, (3) With a history of alcohol or other substance abuse or dependence, (4) Fail to complete scales.

### Clinical measurements

A complete survey was performed using a case report form (CRF). Demographic and clinical data (age, sex, years of education, marital status (married, divorced or widowed), current smoking, and body mass index (BMI)) were documented when the patient was included. Family history of mental disease was also recorded on the CRF. Other clinical variables including age at onset and psychiatric drug equivalent dose were collected.

### Definition of different age group

The SZ patients were divided into two group according to age. The SZ patients aged 18–44 were defined as young SZ group. The SZ patients aged 45–70 were defined as middle-aged and elderly SZ group.

### Psychological assessment

#### Assessment of suicidal ideation

The Beck Scale for Suicide Ideation-Chinese Version (BSI-CV) (www.crisis.org.cn) was used to evaluate the current intensity of suicidal ideation, which was defined as thoughts about ending one’s own life (whether active (with a plan) or passive (with only a wish to die but no specific plan)) [30] among the patients with SZ [31]. Based on their scores for Items 4 or 5 on the Beck Scale for Suicide Ideation, we divided all chronic SZ patients into the SZ with suicidal ideation group and the SZ without suicidal ideation group. A score for Item 4 (desire to make an active suicidal attempt) or Item 5 (passive suicidal desire) of “weak” or “moderate to strong” indicated patients with suicidal ideation, and only when the scores of Item 4 and 5 were definitely “not” was the patient considered to have no suicidal ideation.

#### Assessment of insomnia, psychotic symptoms, cognitive function

The Insomnia Severity Index (ISI) was used to evaluate the severity of insomnia symptoms [32]. The Positive and Negative Syndrome Scale (PANSS) was used to evaluate the severity of psychotic symptoms in SZ patients [33], which is consisted of three domains: positive symptoms, negative symptoms and general symptoms. The PANSS method derives from two established psychiatric rating scales for which interrater agreement and treatment sensitivity have been demonstrated [33]. PANSS has been widely used to assess the psychotic symptoms of SZ patients in China. The Repeatable Battery for the Assessment of Neuropsychological Status (RBANS), including attention, speech, visual span, immediate memory, and delayed memory dimensions, was used to evaluate cognitive function in this study [34]. We used the translated Chinese version of this test, which has established validity and test–retest reliability among patients with SZ and healthy controls [35]. All treatment information was reviewed and confirmed by a panel of experienced psychiatrists. All these scales have been widely used to assess clinical symptoms in patients with SZ. All assessments were performed independently by two professionally trained psychiatrists with a correlation coefficient greater than 0.8.

### Statistical analysis

The results were presented as the means ± standard deviations (SDs) or medians (interquartile ranges) for continuous variables depending on the normal or non-normal distribution of data, while categorical variables were expressed as numbers (percentages). Normally distributed variables were compared using analysis of student’s t test and analysis of variance (ANOVA), while the Mann‒Whitney U test was used for non-normally distributed variables. In addition, variances with significant differences were confirmed by analysis of covariance (ANCOVA). Categorical variables were compared by the chi-squared test. The roles of demographic variables, clinical factors and neurocognitive function in suicidal ideation were checked by stepwise multivariate logistic regression analysis (forward: conditional model) after adjusting the potential risk factors for suicidal ideation. All statistical tests were performed with SPSS for Windows (Release 22.0; SPSS, Chicago, IL, USA). Values of P < 0.05 were considered to be statistically significant in all tests.

## Results

### Demographic characteristics in chronic schizophrenia

A total of 587 patients with chronic SZ were enrolled in our study, which was sufficient after evaluation by G-power. Among 587 SZ patients, the average age of SZ patients was 46.07 ± 12.21 years old, their average years of education were 9.21 ± 3.10 years, and their average age at onset was 25.17 ± 7.55. The average duration of psychosis was 20.90 ± 11.59 years. The prevalence of suicidal ideation reached 23.5% in this study, which represented no significant difference in sex distribution. SZ patients with suicidal ideation had shorter duration of psychosis than SZ patients without suicidal ideation (18.36 ± 10.98 vs. 21.69 ± 11.67, t =-2.974, P = 0.003). There was a negative correlation between the age and attention scores of RBANS in the study (r=-0.099, P = 0.018) (Table 1). However, no correlation was found between age and other distinct cognitive domains in this study (All P > 0.05) (Table 1). Meanwhile, there was a negative correlation between the duration of psychosis and attention scores of RBANS in the study (r=-0.103, P = 0.013). There was a negative correlation between the duration of psychosis and immediate memory scores of RBANS in the study (r=-0.095, P = 0.021). However, no correlation was found between the duration of psychosis and other distinct cognitive domains in this study (All P > 0.05).

**Table 1:** The correlation between age and RBANS

RBANS	Age	
	r	P
Immediate memory scores	-0.067	0.108
Visuospatial scores	-0.003	0.939
Language scores	0.046	0.272
Attention scores	-0.099	0.018*
Delayed memory scores	-0.014	0.729

RBANS, repeatable battery for the assessment of neuropsychological status. Adjusting for education years

*p < 0.05, **p < 0.01

### Demographic characteristics in chronic schizophrenia across age groups

The patients were divided into young SZ group (aged 18–44) and middle-aged and elderly SZ group (aged 45–70). The young SZ group had higher total scores of BSI than middle-aged and elderly SZ group (23.19 ± 20.39 vs. 18.09 ± 17.47, t = 3.201, P = 0.001) (Table 2). Moreover, the prevalence of suicidal ideation in young SZ group was higher than middle-aged and elderly SZ group (30.5% vs. 18.0%, χ 2 = 3.201, P < 0.001) (Table 2). The young SZ group was more likely to have younger age of onset, shorter duration of psychosis and smaller psychiatric drug equivalent dose than middle-aged and elderly SZ group(22.68 ± 5.70 vs. 27.13 ± 8.22, t =-7.731, P < 0.001; 11.86 ± 6.59 vs. 28.05 ± 9.51, t =-24.316, P < 0.001; 266.20 ± 211.71 vs. 389.22 ± 611.42, t =-3.395, P = 0.001)(Table 2).Meanwhile, the young SZ group was less smokers and had higher BMI than middle-aged and elderly SZ group (23.9% vs. 32.6%, χ 2 = 5.323, P = 0.021; 25.17 ± 4.36 vs. 24.40 ± 4.43, t = 2.100, P = 0.036)(Table 2).

**Table 2:** Clinical characteristics in young patients and middle-aged and elderly patients with chronic schizophrenia

	Young patients with SZ(n=259)	Middle-aged and elderly patients with SZ(n=328)	t/Z/χ^2^	P
Gender (female percentage)	95(36.7)	103(31.4)	1.803	0.179
Age(years)	34.54±6.00	55.18±7.09	-38.206	<0.001**
Education year(years)	9.18±3.21	9.03±3.01	-0.183	0.855
Marriage status, n(%)			8.226	0.004**
Unmarried	172(66.3)	186(56.7)		
Married	54(20.8)	76(23.2)		
Divorced	32(12.4)	58(17.7)		
Widowed	1(0.4)	8(2.4)		
Current smoking, n(%)	62(23.9)	107(32.6)	5.323	0.021*
BMI (kg/m^2^)	25.17±4.36	24.40±4.43	2.100	0.036*
Family history of mental diseases, n(%)	51(19.7)	45(13.7)	3.773	0.052
Age of onset(years)	22.68±5.70	27.13±8.22	-7.731	<0.001**
Duration of psychosis(years)	11.86±6.59	28.05±9.51	-24.316	<0.001**
Psychiatric drug equivalent dose (CPZ equivalent mg)	266.20±211.71	389.22±611.42	-3.395	0.001**
ISI	2.97±3.66	2.98±3.49	0.043	0.966
PANSS				
Positive symptoms scores	16.54±5.53	16.06±5.20	1.086	0.278
Negative symptoms scores	20.46±6.71	21.36±6.34	-1.652	0.099
General symptoms scores	40.22±8.70	39.53±8.13	0.994	0.321
The total scores	76(63-89)	76(66-87)	0.204	0.839
RBANS				
Immediate memory scores	58.67±34.76	55.42±14.06	1.411	0.159
Visuospatial scores	78.86±18.21	80.14±17.73	-0.856	0.393
Language scores	79.34±14.16	80.43±12.68	-0.961	0.337
Attention scores	78.75±15.19	77.56±13.81	0.982	0.326
Delayed memory scores	64.46±19.90	64.26±18.25	0.128	0.898
The total scores	65.35±13.40	64.79±12.45	0.523	0.601
The total scores of BSI	23.19±20.39	18.09±17.47	3.201	0.001**
Suicidal ideation	79(30.5)	59(18.0)	12.603	<0.001**

BMI, body mass index; CPZ, chlorpromazine; PANSS, positive and negative syndrome scale; RBANS, repeatable battery for the assessment of neuropsychological status; ISI, insomnia severity index

Note: Normally distributed continuous variables were presented as the means ± standard deviations and were compared using analysis of student’s t test. The test statistic for the student’s t test is “t”. Non-normal distributed continuous variables were presented as medians (interquartile ranges) and were compared using analysis of the Mann‒Whitney U test. The test statistic for the Mann‒Whitney U test is “Z”. Categorical variables were expressed as numbers (percentages) and were compared by the chi-squared test. The test statistic for the chi-squared test is “χ^2^”

*p < 0.05, **p < 0.01

### Demographic characteristics in SZ patients with or without suicidal ideation across age groups

Young SZ patients with suicidal ideation had higher scores of ISI than young SZ patients without suicidal ideation (4.37 ± 4.68 vs. 2.36 ± 2.92, t =-3.530, P = 0.001) (Table 3). Young SZ patients with suicidal ideation had higher total scores of PANSS than young SZ patients without suicidal ideation(80.00(71.00–92.00) vs. 74.50(62.25–87.75), Z =-2.496, P = 0.013), specifically for positive symptoms scores(18.20 ± 6.43 vs. 15.81 ± 4.93, t =-2.948, P = 0.004) and general symptoms scores(42.82 ± 8.84 vs. 39.08 ± 8.41, t =-3.249, P = 0.001)(Table 3). Middle-aged and elderly SZ patients with suicidal ideation had higher scores of ISI than middle-aged and elderly SZ patients without suicidal ideation (4.29 ± 4.40 vs. 2.70 ± 3.19, t =-2.632, P = 0.010) (Table 4). Middle-aged and elderly SZ patients with suicidal ideation had higher total scores of PANSS than middle-aged and elderly SZ patients without suicidal ideation(80.00(70.00–93.00) vs. 75.00(66.00–86.00), Z =-2.021, P = 0.043), specifically for positive symptoms scores(17.69 ± 5.89 vs. 15.70 ± 4.98, t =-2.695, P = 0.007) and general symptoms scores(42.03 ± 8.44 vs. 38.98 ± 7.97, t =-2.638, P = 0.009)(Table 4). Middle-aged and elderly SZ patients with suicidal ideation had higher total scores of RBANS than middle-aged and elderly SZ patients without suicidal ideation(68.47 ± 13.88 vs. 63.97 ± 11.98, t =-2.536, P = 0.012), specifically for visuospatial scores (84.97 ± 19.49 vs. 79.07 ± 17.17, t =-1.975, P = 0.048) and attention scores (81.78 ± 12.62 vs. 76.63 ± 13.91, t =-2.615, P = 0.009)(Table 4).Meanwhile, middle-aged and elderly SZ patients with suicidal ideation had younger age of onset(25.14 ± 7.57 vs. 27.57 ± 8.31, t = 2.069, P = 0.039) and higher BMI (25.54 ± 4.42 vs. 24.15 ± 4.40, t =-2.205, P = 0.028) than middle-aged and elderly SZ patients without suicidal ideation(Table 4). Middle-aged and elderly SZ patients with suicidal ideation were younger than middle-aged and elderly SZ patients without suicidal ideation (53.10 ± 5.55 vs. 55.64 ± 7.31, t = 2.987, P = 0.004) (Table 4).

**Table 3:** Clinical characteristics in young chronic schizophrenia patients with and without suicidal ideation

	Young SZ patients without suicidal ideation(N=180)	Young SZ patients with Suicidal ideation (N=79)	t/Z/χ^2^	P
Gender (female percentage)	64(35.6)	31(39.2)	0.321	0.571
Age(years)	34.68±6.03	34.20±5.96	0.593	0.553
Education year(years)	9.16±2.92	9.24±3.81	-0.177	0.860
Marriage status, n(%)			0.042	0.838
Unmarried	121(67.2)	51(64.6)		
Married	36(20.0)	18(22.8)		
Divorced	22(12.2)	10(12.7)		
Widowed	1(0.6)	0(0)		
Current smoking, n(%)	41(22.8)	21(26.6)	0.436	0.509
BMI (kg/m^2^)	25.07±4.20	25.37±4.73	-0.507	0.617
Family history of mental diseases, n(%)	33(18.3)	18(22.8)	0.688	0.407
Age of onset(years)	22.53±5.56	23.03±6.03	-0.646	0.519
Duration of psychosis(years)	12.16±6.64	11.18±6.45	1.101	0.272
Psychiatric drug equivalent dose (CPZ equivalent mg)	278.14±225.95	239.00±173.25	1.519	0.130
ISI	2.36±2.92	4.37±4.68	-3.530	0.001**
PANSS				
Positive symptoms scores	15.81±4.93	18.20±6.43	-2.948	0.004**
Negative symptoms scores	20.41±6.61	20.58±6.96	-0.189	0.850
General symptoms scores	39.08±8.41	42.82±8.84	-3.249	0.001**
The total scores	74.50(62.25-87.75)	80.00(71.00-92.00)	-2.496	0.013*
RBANS				
Immediate memory scores	56.26±15.21	64.19±58.64	-1.177	0.243
Visuospatial scores	79.80±18.76	76.68±16.80	1.266	0.207
Language scores	79.44±15.09	79.12±11.83	0.186	0.853
Attention scores	78.97±15.08	78.23±15.54	0.359	0.720
Delayed memory scores	63.87±19.76	65.83±20.28	-0.728	0.467
The total scores	65.45±13.76	65.12±12.61	0.185	0.853

BMI, body mass index; CPZ, chlorpromazine; PANSS, positive and negative syndrome scale; RBANS, repeatable battery for the assessment of neuropsychological status; ISI, insomnia severity index

Note: Normally distributed continuous variables were presented as the means ± standard deviations and were compared using analysis of student’s t test. The test statistic for the student’s t test is “t”. Non-normal distributed continuous variables were presented as medians (interquartile ranges) and were compared using analysis of the Mann‒Whitney U test. The test statistic for the Mann‒Whitney U test is “Z”. Categorical variables were expressed as numbers (percentages) and were compared by the chi-squared test. The test statistic for the chi-squared test is “χ2”

*p < 0.05, **p < 0.01

**Table 4:** Clinical characteristics in middle-aged and elderly schizophrenia patients with and without suicidal ideation

	Middle-aged and elderly SZ patients without suicidal ideation (n=269)	Middle-aged and elderly SZ patients with suicidal ideation (n=59)	t/Z/χ^2^	P
Gender (female percentage)	81(30.1)	22(37.3)	1.157	0.282
Age(years)	55.64±7.31	53.10±5.55	2.987	0.004**
Education year(years)	9.14±3.06	9.64±2.76	-1.170	0.243
Marriage status, n(%)			0.281	0.596
Unmarried	153(56.9)	33(55.9)		
Married	65(24.2)	11(18.6)		
Divorced	44(16.4)	14(23.7)		
Widowed	7(2.6)	1(1.7)		
Current smoking, n(%)	88(32.7)	19(32.2)	0.006	0.940
BMI (kg/m^2^)	24.15±4.40	25.54±4.42	-2.205	0.028*
Family history of mental diseases, n(%)	34(12.6)	11(18.6)	1.474	0.225
Age of onset(years)	27.57±8.31	25.14±7.57	2.069	0.039*
Duration of psychosis(years)	28.07±9.82	27.97±8.05	0.074	0.941
Psychiatric drug equivalent dose (CPZ equivalent mg)	382.08±646.63	421.78±417.49	-0.451	0.652
ISI	2.70±3.19	4.29±4.40	-2.632	0.010*
PANSS				
Positive symptoms scores	15.70±4.98	17.69±5.89	-2.695	0.007**
Negative symptoms scores	21.53±6.33	20.56±6.38	1.062	0.291
General symptoms scores	38.98±7.97	42.03±8.44	-2.638	0.009**
The total scores	75.00(66.00-86.00)	80.00(70.00-93.00)	-2.021	0.043*
RBANS				
Immediate memory scores	54.69±13.53	58.69±15.95	-1.791	0.077
Visuospatial scores	79.07±17.17	84.97±19.49	-1.975	0.048*
Language scores	80.47±12.25	80.22±14.56	0.124	0.901
Attention scores	76.63±13.91	81.78±12.62	-2.615	0.009**
Delayed memory scores	63.64±17.81	67.05±20.05	-1.301	0.194
The total scores	63.97±11.98	68.47±13.88	-2.536	0.012*

BMI, body mass index; CPZ, chlorpromazine; PANSS, positive and negative syndrome scale; RBANS, repeatable battery for the assessment of neuropsychological status; ISI, insomnia severity index

Note: Normally distributed continuous variables were presented as the means ± standard deviations and were compared using analysis of student’s t test. The test statistic for the student’s t test is “t”. Non-normal distributed continuous variables were presented as medians (interquartile ranges) and were compared using analysis of the Mann‒Whitney U test. The test statistic for the Mann‒Whitney U test is “Z”. Categorical variables were expressed as numbers (percentages) and were compared by the chi-squared test. The test statistic for the chi-squared test is “χ2”

*p < 0.05, **p < 0.01

### Logistic regression of risk factors for suicidal ideation in SZ patients across age groups

Logistic regression analysis was applied to investigate the risk factors of suicidal ideation among chronic SZ. The role of neurocognitive function in suicidal ideation was evaluated by logistic regression analysis after adjusting for all potential risk factors of suicidal ideation. The results demonstrated that the scores of ISI (Odds ratio(OR), 1.151; 95% Confidence interval(95%CI), [ 1.068–1.241]; P < 0.001), and positive symptoms scores of PANSS(OR, 1.078; 95%CI, [1.026–1.133]; P = 0.003)were associated with suicidal ideation in young SZ patients(Table 5). The results demonstrated that age (OR, 0.952; 95%CI, [0.908–0.997]; P = 0.038), BMI (OR, 1.079; 95%CI, [1.005–1.159]; P = 0.035), the scores of ISI (OR, 1.116; 95%CI, [1.029–1.211]; P = 0.008), general symptoms scores of PANSS (OR, 1.077; 95%CI, [1.031–1.124]; P = 0.001), visuospatial scores of RBANS (OR, 1.021; 95%CI, [1.002–1.040]; P = 0.031) and attention scores of RBANS (OR, 1.034; 95%CI, [1.007–1.061]; P = 0.013) were associated with suicidal ideation in middle-aged and elderly SZ patients(Table 6).

**Table 5:** The risk factors for suicidal ideation in young schizophrenia patients

	OR	95%CI	P
ISI	1.151	1.068-1.241	<0.001**
Positive symptoms scores of PANSS	1.078	1.026-1.133	0.003**

*p < 0.05, **p < 0.01

**Table 6:** The risk factors for suicidal ideation in middle-aged and elderly schizophrenia patients

	OR	95%CI	P
Age	0.952	0.908-0.997	0.038*
BMI (kg/m2)	1.079	1.005-1.159	0.035*
ISI	1.116	1.029-1.211	0.008**
General symptoms scores of PANSS	1.077	1.031-1.124	0.001**
Visuospatial scores of RBANS	1.021	1.002-1.040	0.031*
Attention scores of RBANS	1.034	1.007-1.061	0.013*

*p < 0.05, **p < 0.01

## Discussion

To the best of our knowledge, this is the first study to detect the relationship between neurocognition and suicidal ideation in SZ patients across age groups. The results of this study were as follows: (1) The higher age was related to decreased attention. (2) The young SZ patients had higher risk of suicidality than middle-aged and elderly SZ patients. (3) Young SZ patients with severe insomnia and positive psychotic symptoms were more vulnerable to suicidal ideation. (4) Compared with the middle-aged and elderly SZ patients without suicidal ideation, the middle-aged and elderly SZ patients with suicidal ideation were younger, had higher BMI, had more severe insomnia and general psychotic symptoms, and had better visuospatial ability and attention.

In this study, we found that 23.5% of SZ patients had suicidal ideation, which is consistent with the results of previous studies [25, 36–38].Moreover, we found that young SZ patients had higher risk of suicidality than middle-aged and elderly SZ patients, which is consistent with the previous studies [2, 39].Meanwhile, young age was a risk factor for suicidal ideation in middle-aged and elderly SZ patients. A positive effect of age on mental health-related quality of life (HRQOL) has been found in the previous study [40]. The old SZ patients perceive and adjust to their illnesses well [41], which might be used to explain the low prevalence of suicidal ideation in the middle-aged and elderly patients. Meanwhile, high BMI was found to be a risk factor for suicidal ideation in the middle-aged and elderly patients, which is consistent with the previous study [42].

We found higher score of ISI was associated with the occurrence of suicidal ideation in both young SZ patients and middle-aged and elderly SZ patients. Previous study has reported that higher score of ISI is associated with the occurrence of suicidal ideation in SZ patients [13, 43, 44]. The underlying mechanism of association between insomnia and suicidal ideation in SZ patients is unclear. Several psychological and physiological mechanisms may mediate the association between insomnia and suicidal ideation such as hopelessness, serotonergic dysfunction, and hypothalamic-pituitary adrenal (HPA) axis dysfunction [45]. As we know, chronic insomnia patients are more likely to feel hopeless because of the dysfunctional beliefs and attitudes about sleep [45], which may lead to the occurrence of suicidal ideation [46]. In addition, sleep deprivation of rodents leads to a loss of sensitivity of post-synaptic 5-HT receptors [47, 48], and this serotonin desensitization is paralleled by a blunted hypothalamic-pituitary-adrenal (HPA) axis [47]. Serotonergic dysfunction and hypothalamic-pituitary adrenal (HPA) axis dysfunction are associated with the occurrence of suicidal ideation [49].

We also found that severe positive symptom was associated with suicidal ideation among young SZ patients, while general symptom was associated with suicidal ideation among the middle-aged and elderly patients. The relationship between psychotic symptoms and suicidal ideation was largely inconclusive. A meta-analysis has shown that the positive symptoms, negative symptoms and general symptoms of PANSS are both the risk factors for suicidal ideation in SZ patients [50]. However, some studies have suggested that only increased positive symptoms or decreased negative symptoms are associated with suicidal ideation in SZ patients [51–55]. Meanwhile, general symptoms of PANSS have been proved to be associated with depression and negative expectation [56, 57]. SZ patients with depression have severe positive symptoms, general symptoms of PANSS and high rates of suicidal behavior [56]. Another study has shown that SZ patients with suicidal attempt have severe positive symptoms of PANSS and general symptoms of PANSS, which is consistent with our results [58]. The possible explanations related to this inconclusive finding may be due to the patients in different stage of disease progression (acute vs. chronic or active phase vs. remission), different age or different illness duration, different exposure to antipsychotic treatment (naive vs. medicated). Our study found that the relationship between psychotic symptoms and suicidal ideation in SZ patients was different in different age group. More studies are needed to explore the relationship between psychotic symptoms and suicidal ideation in the future. A conclusion could be drawn according to the results of previous studies that patients will probably develop suicidal intention when their psychotic symptoms are grievous. We speculated that there were two reasons. First, patients may suffer imperative auditory hallucinations or persecutory delusion. Second, a long-term illness may make the patient feel anxious, fearful and desperate.

In this study, visuospatial scores of RBANS and attention scores of RBANS were associated with suicidal ideation in the middle-aged and elderly SZ patients, while no correlation was found between neurocognition and suicidal ideation in young SZ patients. The relationship between neurocognition and suicidal ideation is heterogeneous in previous studies. Some studies insisted that preserved neurocognition is associated with the suicidality [15, 21, 22], while the other studies insisted that there is no correlation between neurocognition and suicidality [23, 24, 59]. There are various reasons for these heterogeneous results, such as patient selection bias, different definitions of suicidal categories and different measurement tools for neurocognition. Meanwhile, the different age of SZ patients included in these studies might be another reason for heterogeneous results. Neurocognitive function is correlated negatively with age in SZ patients [27]. SZ patients have showed greater deteriorated performance with increasing age on attention [29]. We also detected a negative correlation between attention scores of RBANS and age in the study, which is consistent with the previous result. In fact, preserved neurocognition function is necessary in the process of suicidal ideation. Compared to the middle-aged and elderly SZ patients, most young SZ patients usually have better neurocognition. Therefore, the difference of neurocognitive function in young SZ patients is smaller than that in middle-aged and elderly SZ patients. It might explain why neurocognitive function is not related to the occurrence of suicidal ideation in young SZ patients and the effect of neurocognition in suicidal ideation is obviously found in the middle-aged and elderly SZ patients. Visuospatial ability and attention were risk factors for suicidal ideation in middle-aged and elderly SZ patients. The underlying mechanism is unknown. It may be that middle-aged and elderly SZ patients with good visuospatial ability and attention tend to have good insight and execution function. Good insight and execution function have been affirmed to be associated with suicidality [60–63]. The patients with good visuospatial ability and attention will have better insight and awareness of their illness and its debilitating effects [15]. The SZ patients who are awareness of delusions, blunted affect, anhedonia [60], the need for treatment [64, 65], and the social consequences of the disorder [65] would be vulnerable to suicidal ideation and suicide attempt. Executive function is the capacity to shift attention from one stimulus to another, initiate or cease engaging in a given behavior, evaluate risk, and develop plans of action and carry them out [62]. Impairment in this visuospatial and attentional system will result in the difficulties with goal formulation and an inability to plan effectively [34, 62]. Thus, those middle-aged and elderly SZ patients with relatively better visuospatial ability and attention may have greater ability to formulate suicidal ideation [62].

There are several limitations in the study. Firstly, this is a cross-sectional study, and it cannot show direct causality between suicidal ideation and the risk factors in SZ patients. Secondly, the all subjects are inpatients and the selection biases may limit the generalizability of the findings. Thirdly, we have no healthy control group. Fourthly, compared with SCID, Beck SI scale is not the best way to assess the suicidal ideation of SZ patients. Fifthly, considering the complexity of the neurocognition, the result measured by the RBANS may be not particularly perfect and some domains of neurocognition related with suicidal ideation may not be detected. Sixthly, the side effects of antipsychotics are not recorded in this study, which may be associated with suicidal ideation in SZ patients. Seventhly, the female patients who are pregnant, planning to become pregnant, or breastfeeding are not included in the study, which may limit the generalizability of the findings.

## Conclusions

Our study explored the correlation between neurocognition and suicidal ideation in SZ patients across age groups. We found that good visuospatial ability and attention function were associated with the occurrence of suicidal ideation in the middle-aged and elderly SZ patients. No correlation between neurocognition and suicidal ideation was found in young SZ patients. Hence, we should devote additional concern on the middle-aged and elderly SZ with good neurocognition. An effective and new intervention focusing on distracting attention from the disease to reduce the occurrence of suicidal ideation should be formulated. Further cohort studies are warranted to detect the relationship between neurocognition and suicidal ideation across age groups.

## Data Availability

The data that support the study conclusions are available from the corresponding author upon reasonable request.

## References

[1] Charlson FJ, Ferrari AJ, Santomauro DF (2018). Global Epidemiology and burden of Schizophrenia: findings from the global burden of Disease Study 2016. Schizophr Bull.

[2] Olfson M, Stroup TS, Huang C, Wall MM, Crystal S, Gerhard T (2021). Suicide risk in Medicare patients with Schizophrenia across the Life Span. JAMA Psychiatry.

[3] Ventriglio A, Gentile A, Bonfitto I (2016). Suicide in the early stage of Schizophrenia. Front Psychiatry.

[4] Balhara YP, Verma R (2012). Schizophrenia and Suicide. East Asian Arch Psychiatry.

[5] Lopez-Morinigo JD, Ayesa-Arriola R, Torres-Romano B (2016). Risk assessment and Suicide by patients with schizophrenia in secondary mental healthcare: a case-control study. BMJ Open.

[6] Melle I, Johannesen JO, Friis S (2006). Early detection of the first episode of schizophrenia and suicidal behavior. Am J Psychiatry.

[7] Andriopoulos I, Ellul J, Skokou M, Beratis S (2011). Suicidality in the prodromal phase of schizophrenia. Compr Psychiatry.

[8] Li Y, Hou CL, Ma XR (2017). Quality of life in Chinese patients with schizophrenia treated in primary care. Psychiatry Res.

[9] Chan KY, Zhao FF, Meng S (2015). Prevalence of schizophrenia in China between 1990 and 2010. J Glob Health.

[10] Bai W, Liu ZH, Jiang YY (2021). Worldwide prevalence of suicidal ideation and Suicide plan among people with schizophrenia: a meta-analysis and systematic review of epidemiological surveys. Transl Psychiatry.

[11] De Hert M, McKenzie K, Peuskens J (2001). Risk factors for Suicide in young people suffering from schizophrenia: a long-term follow-up study. Schizophr Res.

[12] Chong BTW, Wahab S, Muthukrishnan A, Tan KL, Ch’ng ML, Yoong MT (2020). Prevalence and Factors Associated with suicidal ideation in Institutionalized patients with Schizophrenia. Psychol Res Behav Manag.

[13] Miller BJ, McCall WV, Xia L (2021). Insomnia, suicidal ideation, and psychopathology in Chinese patients with chronic schizophrenia. Prog Neuropsychopharmacol Biol Psychiatry.

[14] Flanagan P, Compton MT (2012). A comparison of correlates of suicidal ideation prior to initial hospitalization for first-episode psychosis with prior research on correlates of Suicide attempts prior to initial treatment seeking. Early Interv Psychiatry.

[15] Delaney C, McGrane J, Cummings E (2012). Preserved cognitive function is associated with suicidal ideation and single Suicide attempts in schizophrenia. Schizophr Res.

[16] Pompili M, Amador XF, Girardi P (2007). Suicide risk in schizophrenia: learning from the past to change the future. Ann Gen Psychiatry.

[17] Silva RMD, Sousa GS, Vieira L, Caldas JMP, Minayo MCS (2018). Suicidal ideation and attempt of older women in Northeastern Brazil. Rev Bras Enferm.

[18] Bohaterewicz B, Sobczak AM, Krzesniak A, Metel D, Adamczyk P (2021). On the relation of gyrification and cortical thickness alterations to the suicidal risk and mental pain in chronic schizophrenia outpatients. Psychiatry Res Neuroimaging.

[19] Sheffield JM, Barch DM (2016). Cognition and resting-state functional connectivity in schizophrenia. Neurosci Biobehav Rev.

[20] Harvey PD, Isner EC, Cognition (2020). Social Cognition, and functional capacity in early-onset Schizophrenia. Child Adolesc Psychiatr Clin N Am.

[21] Zhang XY, Du X, Yin G et al. Prevalence and clinical correlates of and cognitive function at the time of Suicide attempts in first-episode and drug-naive patients with Schizophrenia. J Clin Psychiatry. 2018;79(4). 10.4088/JCP.17m11797 30063302

[22] Huang Y, Wu K, Jiang R (2021). Suicide attempts, neurocognitive dysfunctions and clinical correlates in Middle-aged and Elderly Chinese Schizophrenia patients. Front Psychiatry.

[23] Potkin SG, Anand R, Alphs L, Fleming K (2003). Neurocognitive performance does not correlate with suicidality in Schizophrenic and schizoaffective patients at risk for Suicide. Schizophr Res.

[24] Barrett EA, Sundet K, Simonsen C (2011). Neurocognitive functioning and suicidality in schizophrenia spectrum disorders. Compr Psychiatry.

[25] Ran MS, Wu QH, Conwell Y, Chen EY, Chan CL (2004). Suicidal behavior among inpatients with schizophrenia and mood disorders in Chengdu, China. Suicide Life Threat Behav.

[26] Villa J, Choi J, Kangas JL, Kaufmann CN, Harvey PD, Depp CA (2018). Associations of suicidality with cognitive ability and cognitive insight in outpatients with Schizophrenia. Schizophr Res.

[27] Bellino S, Rocca P, Patria L (2004). Relationships of age at onset with clinical features and cognitive functions in a sample of schizophrenia patients. J Clin Psychiatry.

[28] Eyler Zorrilla LT, Heaton RK, McAdams LA, Zisook S, Harris MJ, Jeste DV (2000). Cross-sectional study of older outpatients with schizophrenia and healthy comparison subjects: no differences in age-related cognitive decline. Am J Psychiatry.

[29] Lee J, Green MF, Nuechterlein KH (2020). The effects of age and sex on cognitive impairment in schizophrenia: findings from the Consortium on the Genetics of Schizophrenia (COGS) study. PLoS ONE.

[30] Turecki G, Brent DA, Gunnell D (2019). Suicide and Suicide risk. Nat Rev Dis Primers.

[31] Beck AT, Steer RA, Ranieri WF (1988). Scale for Suicide ideation: psychometric properties of a self-report version. J Clin Psychol.

[32] Bastien CH, Vallieres A, Morin CM (2001). Validation of the Insomnia Severity Index as an outcome measure for insomnia research. Sleep Med.

[33] Kay SR, Fiszbein A, Opler LA (1987). The positive and negative syndrome scale (PANSS) for schizophrenia. Schizophr Bull.

[34] Randolph C, Tierney MC, Mohr E, Chase TN (1998). The repeatable Battery for the Assessment of Neuropsychological Status (RBANS): preliminary clinical validity. J Clin Exp Neuropsychol.

[35] Hui L, Han M, Huang XF (2016). Association between DbetaH 5’-insertion/deletion polymorphism and cognition in patients with chronic schizophrenia. J Clin Psychiatry.

[36] Minzenberg MJ, Lesh TA, Niendam TA, Yoon JH, Rhoades RN, Carter CS (2014). Frontal cortex control dysfunction related to long-term Suicide risk in recent-onset schizophrenia. Schizophr Res.

[37] Fenton WS, McGlashan TH, Victor BJ, Blyler CR (1997). Symptoms, subtype, and suicidality in patients with schizophrenia spectrum disorders. Am J Psychiatry.

[38] Radomsky ED, Haas GL, Mann JJ, Sweeney JA (1999). Suicidal behavior in patients with schizophrenia and other psychotic disorders. Am J Psychiatry.

[39] Okusaga OO, Kember RL, Peloso GM (2021). History of Suicide attempts and COVID-19 Infection in veterans with Schizophrenia or Schizoaffective Disorder: moderating effects of Age and Body Mass Index. Complex Psychiatry.

[40] Folsom DP, Depp C, Palmer BW (2009). Physical and mental health-related quality of life among older people with schizophrenia. Schizophr Res.

[41] Shepherd S, Depp CA, Harris G, Halpain M, Palinkas LA, Jeste DV (2012). Perspectives on schizophrenia over the lifespan: a qualitative study. Schizophr Bull.

[42] Cheng P, Ju P, Xia Q (2022). Childhood maltreatment increases the suicidal risk in Chinese schizophrenia patients. Front Psychiatry.

[43] Miller BJ, McEvoy JP, McCall WV, Insomnia. Suicidal ideation, and Suicide attempts in the clinical antipsychotic trials of intervention effectiveness. J Clin Psychiatry. 2021;82(3). 10.4088/JCP.20m13338 34033271

[44] Miller BJ, Parker CB, Rapaport MH, Buckley PF, McCall WV. Insomnia and suicidal ideation in nonaffective psychosis. Sleep. 2019;42(2). 10.1093/sleep/zsy215 PMC6369728 30407600

[45] McCall WV, Black CG (2013). The link between Suicide and insomnia: theoretical mechanisms. Curr Psychiatry Rep.

[46] Simpson GK, Tate RL, Whiting DL, Cotter RE (2011). Suicide prevention after traumatic brain injury: a randomized controlled trial of a program for the psychological treatment of hopelessness. J Head Trauma Rehabil.

[47] Novati A, Roman V, Cetin T (2008). Chronically restricted sleep leads to depression-like changes in neurotransmitter receptor sensitivity and neuroendocrine stress reactivity in rats. Sleep.

[48] Roman V, Walstra I, Luiten PG, Meerlo P (2005). Too little sleep gradually desensitizes the serotonin 1A receptor system. Sleep.

[49] Pompili M, Serafini G, Innamorati M (2010). The hypothalamic-pituitary-adrenal axis and serotonin abnormalities: a selective overview for the implications of Suicide prevention. Eur Arch Psychiatry Clin Neurosci.

[50] Cassidy RM, Yang F, Kapczinski F, Passos IC (2018). Risk factors for suicidality in patients with Schizophrenia: a systematic review, Meta-analysis, and Meta-regression of 96 studies. Schizophr Bull.

[51] De Sousa A, Shah B, Shrivastava A (2020). Suicide and Schizophrenia: an interplay of factors. Curr Psychiatry Rep.

[52] Kjelby E, Sinkeviciute I, Gjestad R (2015). Suicidality in schizophrenia spectrum disorders: the relationship to hallucinations and persecutory delusions. Eur Psychiatry.

[53] Yan F, Xiang Y-T, Hou Y-Z (2013). Suicide attempt and suicidal ideation and their associations with demographic and clinical correlates and quality of life in Chinese schizophrenia patients. Soc Psychiatry Psychiatr Epidemiol.

[54] Krynicki CR, Upthegrove R, Deakin JFW, Barnes TRE (2018). The relationship between negative symptoms and depression in schizophrenia: a systematic review. Acta Psychiatr Scand.

[55] Grover LE, Jones R, Bass NJ, McQuillin A (2022). The differential associations of positive and negative symptoms with suicidality. Schizophr Res.

[56] Rajkumar RP (2015). Depressive symptoms during an Acute Schizophrenic Episode: frequency and clinical correlates. Depress Res Treat.

[57] Kao YC, Liu YP, Lu CW (2012). Beck hopelessness scale: exploring its dimensionality in patients with schizophrenia. Psychiatr Q.

[58] Demirkol ME, Tamam L, Namli Z, Karaytug MO, Ugur K (2019). Association of Psychache and Alexithymia with Suicide in patients with Schizophrenia. J Nerv Ment Dis.

[59] Ran MS, Xiang MZ, Mao WJ (2005). Characteristics of Suicide attempters and nonattempters with schizophrenia in a rural community. Suicide Life Threat Behav.

[60] Amador XF, Friedman JH, Kasapis C, Yale SA, Flaum M, Gorman JM (1996). Suicidal behavior in schizophrenia and its relationship to awareness of Illness. Am J Psychiatry.

[61] Tsuang MT (1978). Suicide in schizophrenics, manics, depressives, and surgical controls. A comparison with general population Suicide mortality. Arch Gen Psychiatry.

[62] Nangle JM, Clarke S, Morris DW (2006). Neurocognition and suicidal behaviour in an Irish population with major psychotic disorders. Schizophr Res.

[63] Verma D, Srivastava MK, Singh SK, Bhatia T, Deshpande SN (2016). Lifetime Suicide intent, executive function and insight in schizophrenia and schizoaffective disorders. Schizophr Res.

[64] Schwartz RC, Petersen S (1999). The relationship between insight and suicidality among patients with schizophrenia. J Nerv Ment Dis.

[65] Schwartz RC (2000). Insight and suicidality in schizophrenia: a replication study. J Nerv Ment Dis.

